# Medical Opinion and Movement

**Published:** 1907-08-31

**Authors:** 


### Medical Opinion and Movement.

Whether the great increase in cases of appendicitis is real and due to some factor of modern methods of feeding and living, or apparent only, and due to greater skill in diagnosis, still remains an interesting question for debate. Mr. Battle is fully convinced that there is a very real increase in the incidence of the disease, and has for some time set about to discover the cause, and now offers what appears to be at any rate a plausible solution of the problem. A considerable proportion of cases of appendicitis are associated with appendicular concretions. These concretions were supposed at one time to be "foreign bodies," but careful examination has shown that they are usually masses of fæcal matter combined with inspissated mucus. At the same time, as Mr. Battle points out, these concretions are frequently formed around a foreign body as a nucleus, which may be quite minute. In a case of appendicitis operated upon by Mr. Battle, a concretion was found to contain a small irregular fragment of iron, and Mr. Battle suggests that minute particles of iron finding their way into the flour milled by fluted steel rollers may account for this increase in appendicitis.

It is not necessary that these particles should always form concretions in order to set up appendicitis. By contact with the mucous membrane they may cause abrasions, and by reflex muscular contraction due to the irritation set up they would probably be washed on again by the fluid contents of the bowel. The abrasion thus formed would give the bacteria their footing, and set up inflammation leading to fibrosis and stricture. Such is briefly the course of events pictured by Mr. Battle. He points out that the greater prevalence of appendicitis was first heard of in the United States, and only later when so much of our flour came from over the seas, and when our own stone mills could no longer be worked at a profit, was there any apparent increase of the disease in this country. Moreover, in America the increase in appendicitis occurred first in the towns where rolled flour was first used, then it spread to the villages, and finally to the negroes, when flour became so cheap that it was more profitable to buy it than to grind the corn at home. Should further investigation support this theory, it would become a question of public importance that proper methods should be adopted to secure the elimination of all iron particles from the flour.

The discussion which took place at the recent meeting of the British Medical Association in the gynæcological section on the early diagnosis of carcinoma uteri deserves the careful consideration of all medical practitioners. In the opening address by Dr. Herbert Spencer it was pointed out that there was a greater mortality among women from cancer of the uterus than from malignant disease of any other part. This he attributed entirely to the fact that cases did not come into the hands of the surgeon at a sufficiently early stage. In spite of the pessimism of certain gynæcologists, he maintained that the successful results hitherto obtained fully justified the belief that cancer of the uterus is as curable as the disease elsewhere, if only it was treated promptly as soon as it was recognisable. The speakers who followed fully concurred in the views expressed by Dr. Spencer, and it was generally agreed that the ignorance of women as to the significance of the usual symptoms of the disease, such as irregular discharges, was chiefly responsible for the present state of affairs. The general practitioner, however, did not escape criticism, and several instances were cited in which it was claimed that fatal delay had been caused by the medical attendant failing to realise the gravity of the condition. In any case the discussion should serve to kindle the whole profession to greater efforts in the early detection of the disease. The earnestness of the section on the question is shown by the resolution which was carried at the close of the discussion, to the effect that the Council of the British Medical Association should be requested to appoint a committee to consider the best means of disseminating knowledge of the importance of the early recognition of uterine cancer.

The pathology and treatment of sleeping sickness have occupied such a prominent position in current medical literature that we need hardly apologise for again referring to the subject in these columns. In his most instructive Harben lectures Professor Ehrlich deals with his theory of atrepsy and atreptic immunity and its practical bearing on the treatment of trypanosomiasis with different chemical substances. These substances which have hitherto proved efficient in combating trypanosomic affections he divides into three groups: (1) Basic triphenylmethane dyes, (2) benzidine dyes, (3) arsenic compounds with (4) salts of mercury as adjuvants. By researches carried out with Drs. Röhl and Browning, Professor Ehrlich has found that by prolonged treatment of the host with any of these groups of chemicals it is possible to obtain strains of trypanosomes which are resistant to the particular group employed. Such resistance is specific for any particular group, extends to different substances of the same group, and persists through generations. He has cultivated his atoxyl-fast race through 125 generations without finding any decrease in its powers of resistance. These facts are of immense importance in regard to practical therapeutics. They suggest that a remedy may fail to effect a cure in any particular case after a certain length of treatment, but at the same time they show that the disease may be combated by combining several remedies. While a powerful trypanocidal remedy might fail to kill all the organisms present, even when administered in almost lethal doses, a combination with a weaker drug might effect an otherwise unattainable cure. Professor Ehrlich further points out that by using races of trypanosomes which have been rendered resistant to known remedies, it will be possible to establish new groups of trypanocidal substances. Such methods open up quite a new field of pharmacological research.

